# A new glioma grading model based on histopathology and Bone Morphogenetic Protein 2 mRNA expression

**DOI:** 10.1038/s41598-020-75574-9

**Published:** 2020-10-28

**Authors:** Kaijia Zhou, Zheng Zhao, Shouwei Li, Yanwei Liu, Guanzhang Li, Tao Jiang

**Affiliations:** 1 Beijing Neurosurgical Institute, Capital Medical University, Beijing, 100070 China; 2 Department of Neurosurgery, Sanbo Brain Hospital, Capital Medical University, Beijing, 100070 China; 3 Department of Neurosurgery, Beijing Tiantan Hospital, Capital Medical University, Beijing, 100070 China; 4 Center of Brain Tumor, Beijing Institute for Brain Disorders, Beijing, 100070 China; 5 China National Clinical Research Center for Neurological Diseases, Beijing, 100070 China

**Keywords:** Predictive markers, Prognostic markers, Neurological disorders, Surgical oncology

## Abstract

Glioma, the most common form of primary malignant brain tumors, is graded based solely on histopathological appearance, which has led to prognostic discrepancies. This study aimed to establish a new glioma grading model by analyzing the expression of Bone Morphogenetic Protein 2 (BMP2) mRNA in patients with gliomas as well, named the Histopathological-BMP2 (HB) system. Clinical information was collected from 692 patients from the Chinese Glioma Genome Atlas database. According to pathological glioma subtypes and the expression of BMP2 mRNA in tumor tissues, the new subtypes HBs, HBh, HBm and HB1 were established, with BMP2 expression highest in HBs and lowest in HB1. Survival periods were analyzed. Based on this, the expression of three BMP2 receptors (BMPR1A, BMPR1B, and BMPR2) was also analyzed, which was related to the prognosis of patients. This new classification model was validated in further groups of patients from the CGGA database (n = 291) and the Cancer Genome Atlas (n = 625). A new glioma grade (HB grade) based on histopathology and BMP2 expression can predict the prognosis of glioma patients, with BMPR1B and BMPR2 expression indicating a different prognosis in different types of gliomas. The higher the concentration of BMP2, the better the prognosis of patients.

## Introduction

Gliomas, the most common primary malignant brain tumor, show high recurrence and mortality rates^1–4^. Initially, they were categorized into grades I to IV based on the appearance of certain histopathologic characteristics, such as vascular proliferation, mitosis, polymorphism and necrosis^5^. However, these diagnostic criteria were subjective and the consistency among different neuropathologists was poor^6^. Further to this, the prognosis differed between similarly graded gliomas. Therefore, the detection of molecular markers in gliomas is also required to provide more information for prognostic judgment and choice of postoperative treatment in addition to the presentation of certain histopathologic characteristics^7,8^. According to the 2016 World Health Organization (WHO) Classification of Tumors of the Central Nervous System (CNS), molecular markers such as mutations within isocitrate dehydrogenase 1 (*IDH1*) and 1p/19q are included in the pathological diagnosis of gliomas^9^, which illustrates the importance of the consideration of molecular characteristics as well.

Bone Morphogenetic Protein (BMP) is a member of the transforming growth factor-β (TGF-β) family. BMP ligands and receptors mediate multiple processes throughout neural development, including the survival, proliferation, morphogenesis, lineage commitment, differentiation and apoptosis of neural stem cells in the CNS^10,11^. Among them, signal transduction of BMP2 plays an important role in the development of many kinds of tumors. BMP2 signal transduction is therefore considered as a potential therapeutic target for glioblastoma because BMP2 can induce differentiation and apoptosis of tumor cells^12,13^. In this study, we reclassified glioma patients based on both histopathological characteristics and BMP2 messenger RNA (mRNA) expression and verified the predictive value of survival and prognosis of this classification model. At the same time, we used this classification model to explore the mechanism of interaction between BMP2 and its receptors in different grades of gliomas.

## Results

### Model establishment

1. General situation of patients with different histopathological types (Table 1).

**Table 1:** Clinical characteristics of the patients included within the study.

variable	N (692)	Sex (female/male)	Age (year)	IDH1 mutation (yes/no/NA)	1p19q LOH (yes/no/NA)	Mean BMP2
O	23	11/12	39.39 ± 2.55	18/1/4	15/3/5	18.93 ± 3.51
OA	77	31/46	38.73 ± 1.11	55/17/5	23/48/6	13.71 ± 1.47
A	38	15/23	38.74 ± 1.65	23/12/3	4/28/6	8.84 ± 1.64
rO	7	2/5	48.86 ± 3.35	6/1/0	5/2/0	28.40 ± 12.71
rA	26	6/20	38.46 ± 1.30	17/5/4	3/23/0	9.09 ± 2.38
rOA	17	7/10	38.65 ± 2.24	16/1/0	8/8/1	21.52 ± 4.90
AO	28	15/13	42.07 ± 1.88	17/4/7	16/9/3	17.09 ± 3.29
AA	34	12/22	41.41 ± 2.01	18/14/2	4/29/1	9.58 ± 1.45
rAO	23	16/7	41.78 ± 2.30	18/4/1	16/7/0	24.35 ± 4.51
rAA	31	16/15	42.97 ± 1.48	23/6/2	29/2/0	9.04 ± 1.44
AOA	82	40/42	40.60 ± 1.21	53/18/11	24/49/9	11.48 ± 1.40
rAOA	57	21/36	39.28 ± 1.42	42/13/2	14/38/5	12.23 ± 1.75
GBM	140	55/85	52.23 ± 1.11	24/109/7	5/108/27	4.89 ± 0.83
rGBM	109	50/59	44.44 ± 1.30	25/81/3	4/97/8	5.55 ± 1.01
Total	692	297/395	43.30 ± 0.47	355/286/51	170/451/71	10.41 ± 0.52

*IDH* isocitrate dehydrogenase 1, *NA* not applicable, *LOH* loss of heterozygosity, *BMP2* Bone Morphogenetic Protein 2, *O* oligodendrocytoma, *OA* oligoastrocytoma, *a* astrocytoma, *rO* recurrent oligodendrocytoma, *rA* recurrent astrocytoma, *rOA* recurrent oligodendrocytoma, *AO* anaplastic oligodendrocytoma, *AA* anaplastic astrocytoma, *rAO* recurrent anaplastic oligodendrocytoma, *rAA* recurrent anaplastic astrocytoma, *AOA* anaplastic oligodendrocytoma, *rAOA* recurrent anaplastic oligoastrocytoma, *GBM* glioblastoma.

Table 1 depicts the data, including the demographics and the presence or absence of particular, defining genetic traits, of the 692 patients included in our study. In different grades of gliomas, the expression of BMP2 mRNA in IDH1-mutated gliomas was higher than that in wild-type gliomas (t = 15.374, *p* = 0.000), and the expression of BMP2 mRNA in 1p19q loss of heterozygosity status was higher than that in the intact (t = 16.329, *p* = 0.000). Besides, the expression of BMP2 mRNA was related to age (t = 4.054, *p* = 0.000). The older patients were, the lower expression BMP2 mRNA showed. However, BMP2 mRNA expression was not related to gender (t = 1.469, *p* = 0.142).
2. Expression of BMP2 in patients with different histopathological types of gliomas (Fig. 1).

**Figure 1:**
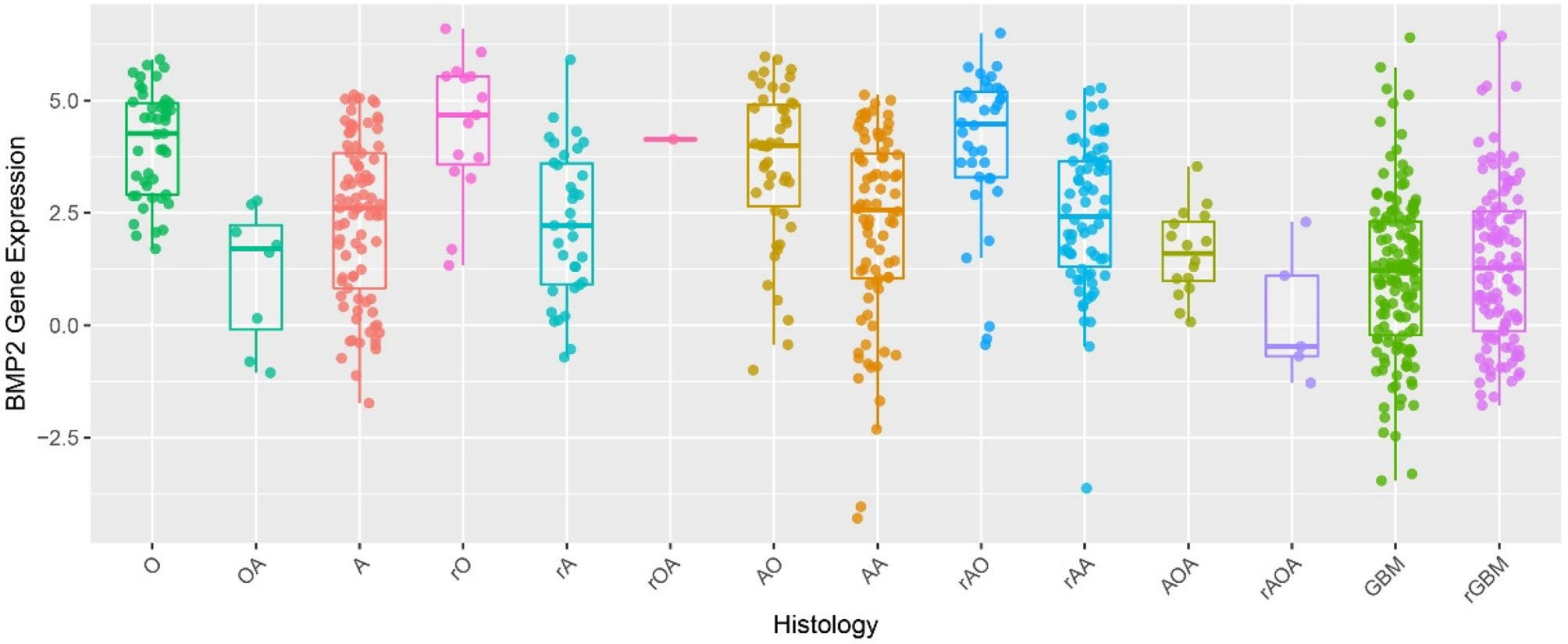
mRNA expression of Bone Morphogenetic Protein 2 (BMP2) in patients with different histopathological types of gliomas. The mRNA expression levels of BMP2 (presented on the y-axis) were collected from patients (n = 692) from the Chinese Glioma Genome Atlas. This cohort was subdivided into 14 groups (n = 7–140) based on the standard classification system which grades gliomas via the histological features observed within stained sections of the tumor (presented on the x-axis). Comparisons were made using one-way ANOVA and data are presented as mean ± standard error.
  The expression of BMP2 was presented against the glioma subtypes defined from histopathological findings in Fig. 1. As can be visualized, BMP2 expression varies with each subtype. Further analysis is detailed below.
3. Mean value curve of BMP2 expression in patients with different histopathological types of gliomas (Fig. 2).

**Figure 2:**
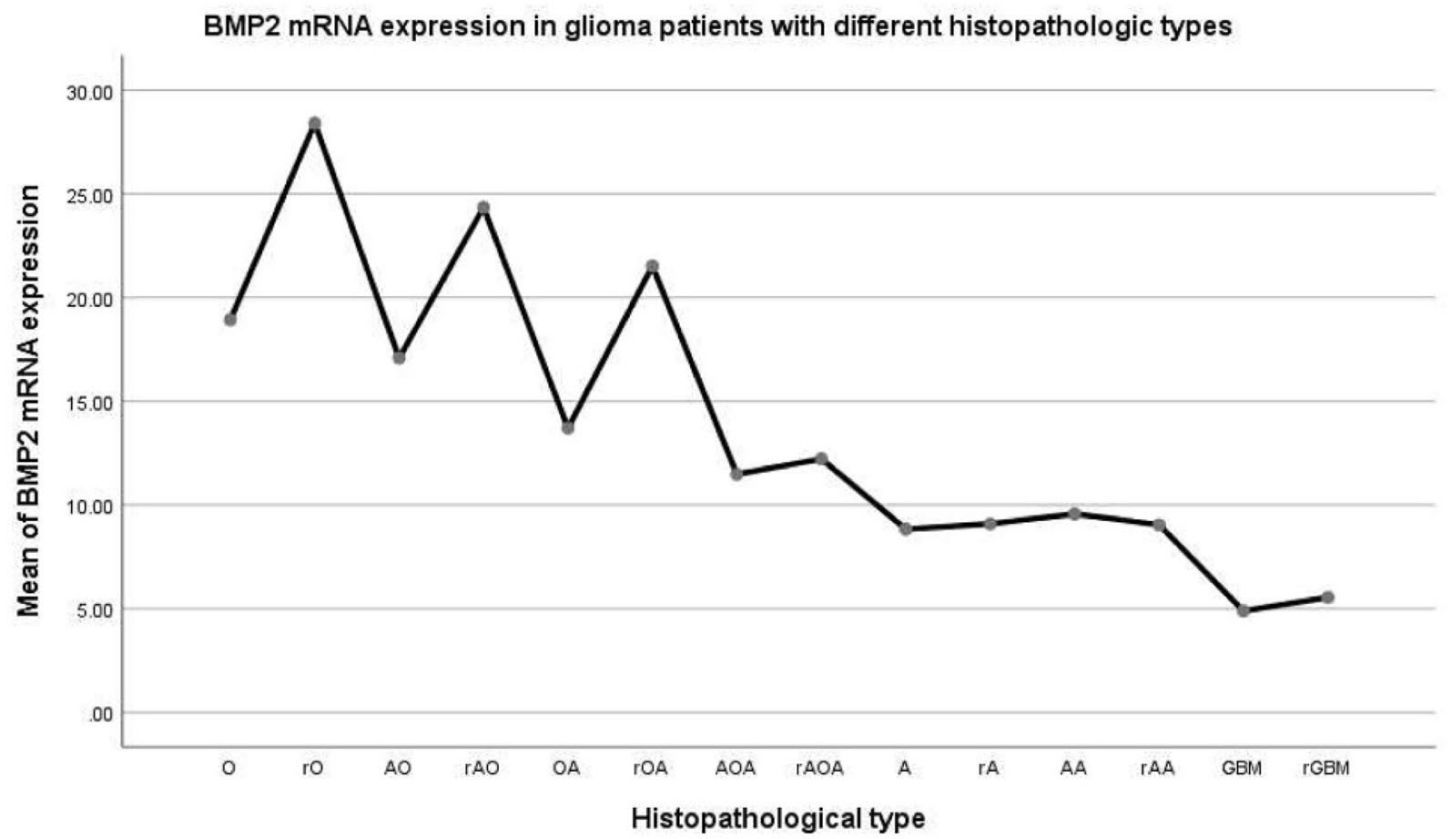
The average mRNA expression of Bone Morphogenetic Protein 2 (BMP2) in different histopathological types of glioma.
  Mean BMP2 expression was observed to differ significantly between the histopathological subtypes, classified via the standard method. The difference of variance analysis was statistically significant: F = 9.392, *p* = 0.000. As can be seen in Fig. 2, the oligodendroma subtypes had the highest BMP2 expression, followed by the astrocytoma subtypes, with the lowest observed in GBM. A pairwise comparison was carried out. Before and after recurrence: O ~ rO increased (*p* = 0.087), AO ~ rAO increased (*p* = 0.023), OA ~ rOA increased (*p* = 0.044); AOA ~ rAOA (*p* = 0.735), A ~ rA (*p* = 0.937), AA ~ rAA (*p* = 0.867) and GBM ~ rGBM (*p* = 0.688) had no change. This suggests that BMP2 increased after glioma recurrence with oligodendrites. BMP2 did not increase after the recurrence of gliomas containing astrocytes or GBM.
  Following trend analysis, there was a significant difference in every two groups in O, rO, AO, rAO, OA, rOA, AOA, rAOA, A, rA, AA, rAA, GBM, and rGBM (F = 9.392, *p* = 0.000). There was no statistical significance in O, AO, and OA. O ~ AO (*p* = 0.608), O ~ OA (*p* = 0.086), AO ~ OA (*p* = 0.231). There was no statistical significance in rO, rAO, and rOA. rO ~ rAO (*p* = 0.463), rO ~ rOA (*p* = 0.231), rAO ~ rOA (*p* = 0.488). There was no significant difference in AOA, rAOA, A, rA, AA and rAA (*p* > 0.05). There was no statistical significance between GBM and rGBM (*p* = 0.688).
4. Patients with gliomas were classified into four grades on the basis of both histopathological characteristics and the expression of BMP2 mRNA.
  Due to the above results, we then re-classified the gliomas based on our findings as outlined below:
  HBs (hispathologic-BMP2 very high expression): rO, rAO, rOA;
  HBh (hispathologic-BMP2 high expression): O, AO, OA;
  HBm (hispathologic-BMP2 middle expression): AOA, rAOA, A, rA, AA, rAA;
  HBl (hispathologic-BMP2 low expression): GBM, rGBM
5. Clinical characteristics of the patients based on HB classification (Table 2).

**Table 2:** Clinical characteristics of the patients based on HB classification.

HB	n	Age (year)	IDH1 mutation (yes/no/NA)	1p19q LOH (yes/no/NA)	Mean BMP2
HBs	47	41.70 ± 1.53	40/6/1	29/17/1	23.93 ± 3.32
HBh	128	39.56 ± 0.91	90/22/16	54/60/14	15.39 ± 1.31
HBm	268	40.22 ± 0.63	176/68/24	48/196/24	10.51 ± 0.70
HBl	249	40.22 ± 0.63	49/190/10	13/205/31	5.18 ± 0.64
Total	692	43.30 ± 0.47	355/286/51	144/478/70	10.41 ± 0.52
  Table 2 represents the patient data following this novel classification system. The difference of variance analysis was statistically significant: F = 38.021, *p* = 0.000. HBs > HBh > HBm > HBl.
6. Mean curve of BMP2 expression in gliomas of different HB grades (Fig. 3).

**Figure 3:**
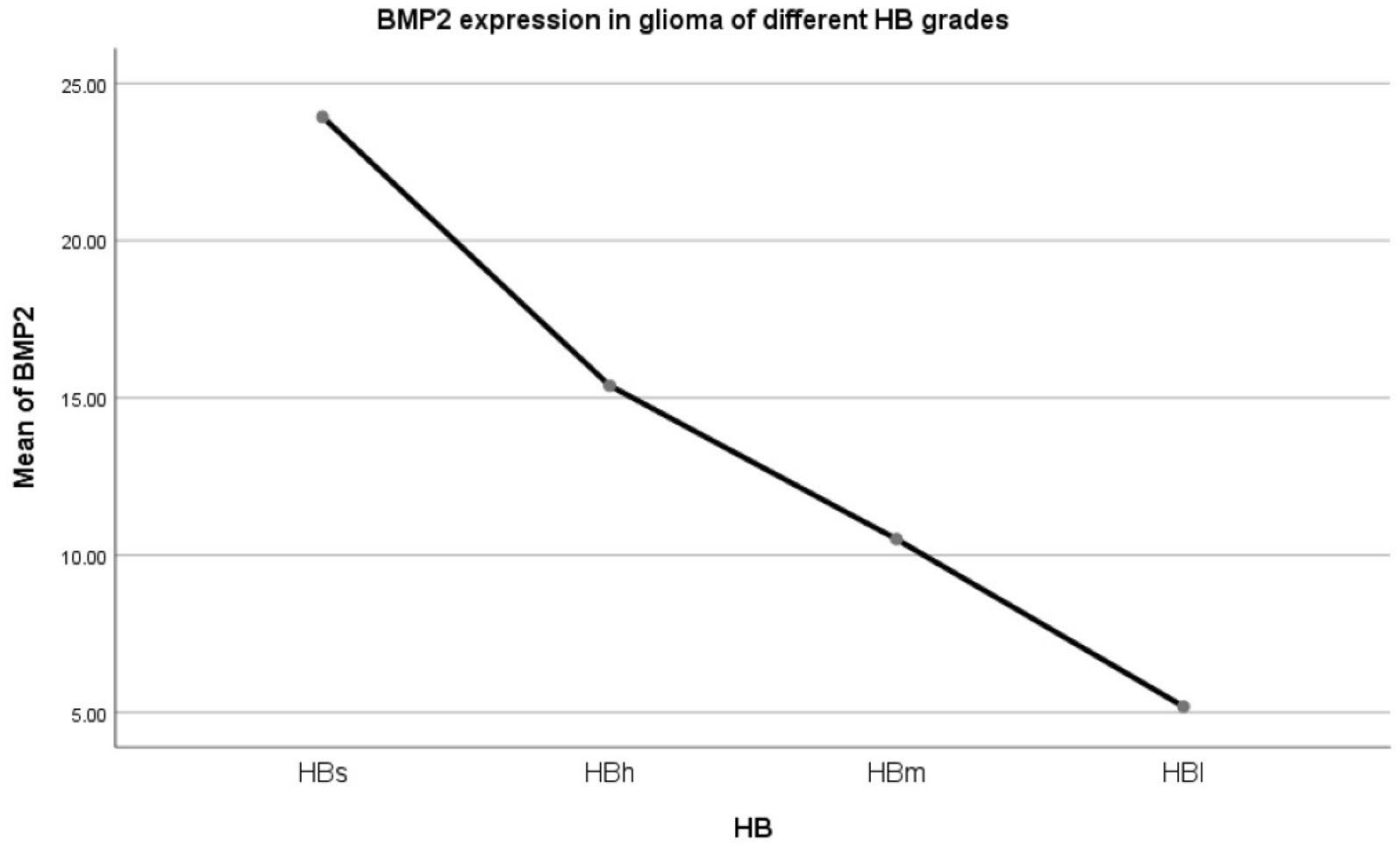
Mean curve of Bone Morphogenetic Protein 2 (BMP2) expression in different HB grade glioma.
  Figure 3 illustrates how BMP2 expression changes throughout these new glioma subtypes, with the highest expression in subtype HBs, as described above. The difference of variance analysis was statistically significant: F = 38.021, *p* = 0.000. Pairwise Comparison: There was a significant difference. (*p* = 0.000).
7. Kaplan–Meier survival curve of glioma patients with different HB grades
  1. Kaplan–Meier (K–M) survival curve of four grades of gliomas (HBs, HBh, HBm, HBl) (Fig. 4).

**Figure 4:**
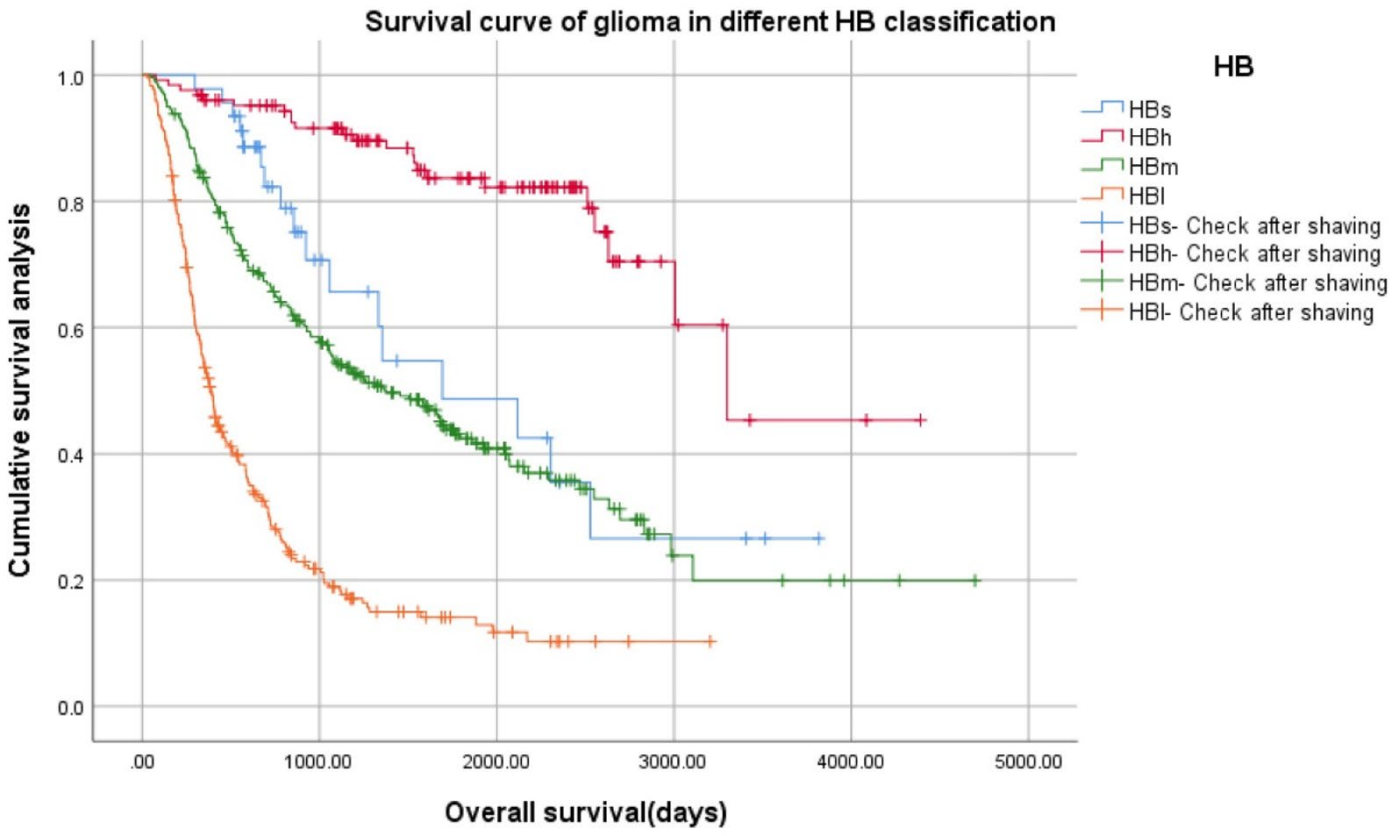
Kaplan–Meier survival curve of four grades of gliomas using the novel proposed HB classification system (HBs, HBh, HBm, HBl).
    Survival of the four glioma subtypes, based on the proposed classification system, was significantly different. Log Rank (Mantel–Cox): x^2^ = 225.112, *p* = 0.000, 24 cases with incomplete follow-up data were excluded from 692 cases. Therefore, 46 cases of HBs, 126 cases of HBh, 259 cases of HBm, and 237 cases of HBl were 668 cases.
  2. K–M survival curve of three grades of gliomas (HBh, HBm, HBl) (Fig. 5).

**Figure 5:**
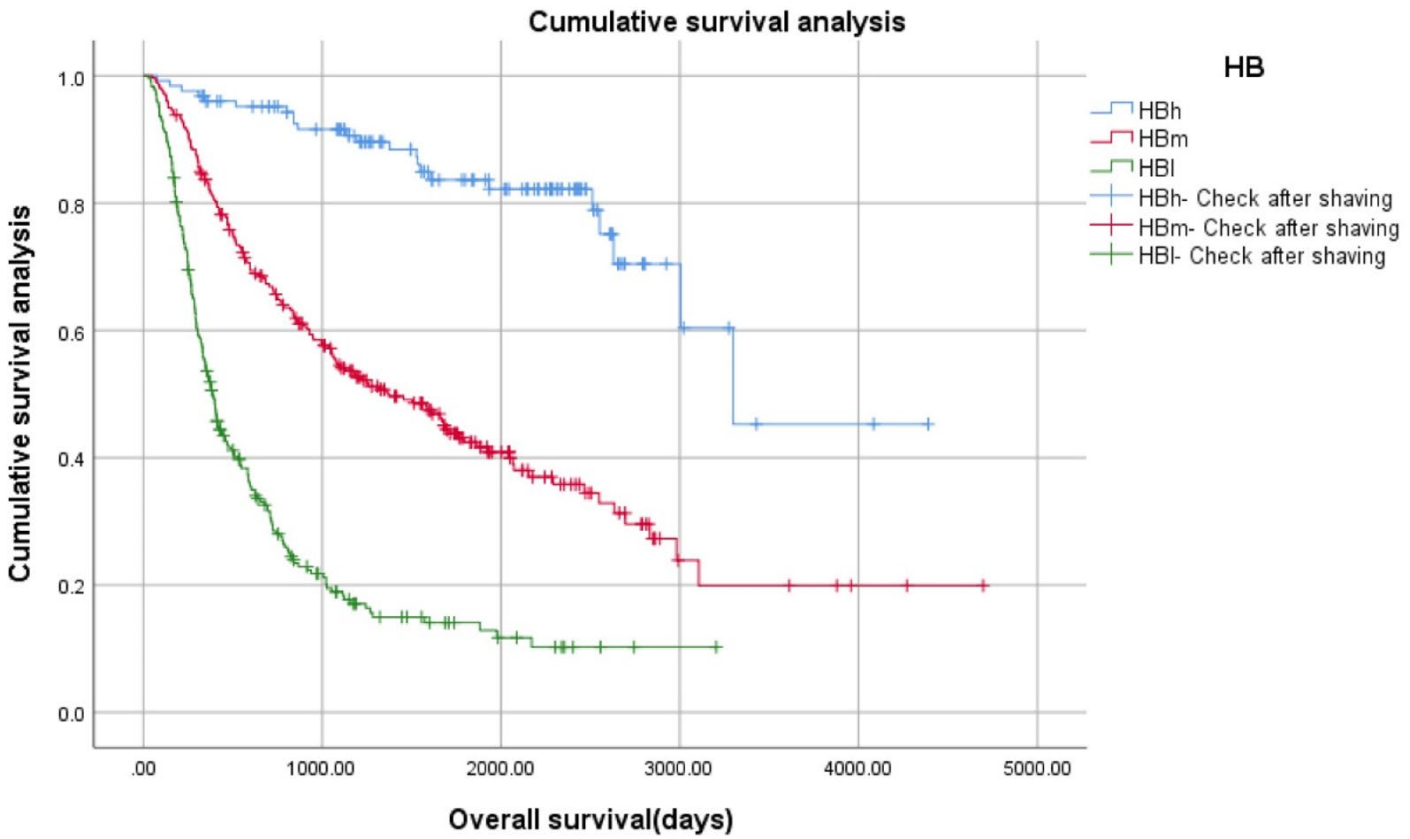
Kaplan–Meier survival curve of three grades of gliomas using the proposed HB classification system (HBh, HBm, HBl).
    Log Rank (Mantel–Cox): x^2^ = 205.419, *p* = 0.000, 126 cases of HBh, 259 cases of HBm, 237 cases of HBl, a total of 622 cases.
  3. K–M survival curve of two grades of gliomas (HBs, HBm) (Fig. 6).

**Figure 6:**
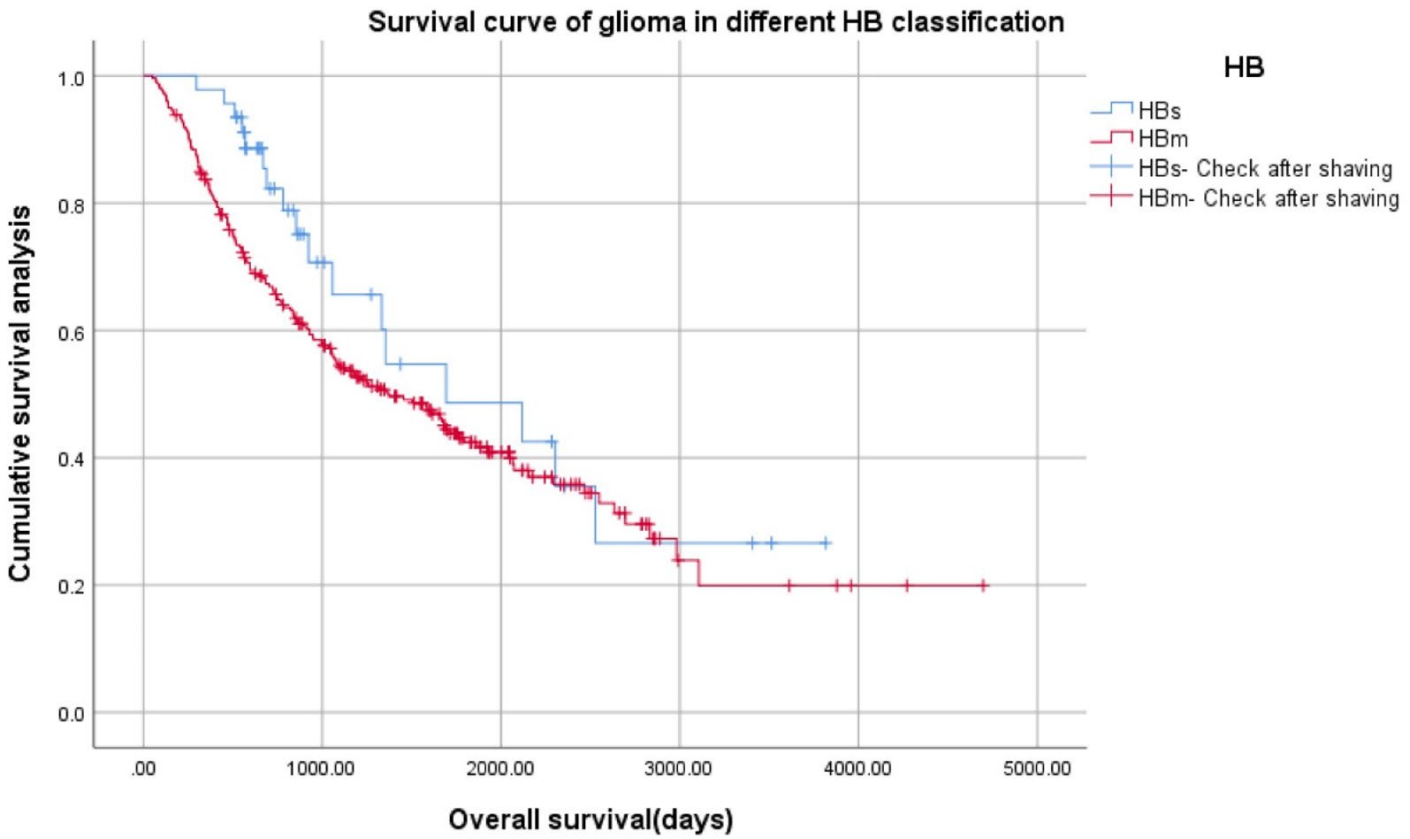
Kaplan–Meier survival curve of two grades of gliomas using the proposed HB classification system (HBs, HBm).
    Log Rank (Mantel–Cox) x^2^ = 1.360, *p* = 0.243, 46 cases of HBs, 259 cases of HBm, a total of 303 cases.

### Model validation

1. Validation group 1: 291 patients from CGGA database.
  In order to validate our proposed classification system, a separate group of patients were analyzed with the above parameters.
  1.1 General condition of 291 glioma patients with different histopathological types in the CGGA verification group (Table 3)

**Table 3:** Clinical characteristics of 291 patients.

variable	n	Sex (female/male	Age (year)	IDH1 mutation (yes/no/NA)	1p19qLOH (yes/no/NA)	Mean BMP2
O	26	8/18	40.65 ± 1.39	23/3/0	22/4/0	28.24 ± 3.48
OA	35	16/19	37.62 ± 1.37	33/2/0	22/13/0	24.65 ± 2.54
A	33	13/20	38.85 ± 1.80	27/5/1	0/32/1	16.66 ± 2.19
rO	0	0	0	0/0/0	0/0/0	0
rA	6	4/2	41.00 ± 5.77	4/2/0	0/6/0	9.85 ± 3.36
rOA	3	0/3	44.33 ± 3.76	3/0/0	0/3/0	14.76 ± 5.44
AO	9	4/5	48.00 ± 3.53	8/1/0	6/2/1	24.03 ± 4.21
AA	14	2/12	47.79 ± 3.82	3/11/0	0/14/0	8.25 ± 2.89
rAO	3	3/0	41.67 ± 1.33	3/0/0	2/1/0	19.67 ± 6.48
rAA	14	7/7	37.29 ± 2.16	7/7/0	1/13/0	7.36 ± 2.15
AOA	27	12/15	40.70 ± 2.86	11/16/0	4/23/0	11.36 ± 2.77
rAOA	12	5/7	36.25 ± 2.55	11/1/0	3/9/0	28.66 ± 8.86
GBM	85	32/53	49.08 ± 1.35	11/74/0	0/84/1	6.35 ± 0.87
rGBM	24	10/14	47.29 ± 2.23	9/15/0	3/21/0	7.17 ± 1.82
Total	291	116/175	43.38 ± 0.70	153/137/1	63/225/3	14.11 ± 0.90
  1.2 Clinical characteristics of 291 patients based on HB classification (Table 4).

**Table 4:** Clinical characteristics of 291 patients based on HB classification.

HB	n	Age (year)	IDH1 mutation (yes/no/NA)	1p19qLOH (yes/no/NA)	Mean BMP2
HBs	3	44.33 ± 3.76	3/0/0	0/3/0	14.76 ± 5.44
HBh	73	40.15 ± 0.99	67/6/0	52/20/1	25.65 ± 1.82
HBm	106	40.12 ± 1.19	63/42/1	8/97/1	13.94 ± 1.58
HBl	109	48.69 ± 1.16	20/89/0	3/105/1	6.53 ± 0.79
TOTAL	291	43.38 ± 0.70	153/137/1	63/225/3	14.11 ± 0.90
    Table 4 depicts the demographics and clinical characteristics, such as the presence of histopathological markers and genetic characteristics, of the patients included in the validation group.
  1.3 Mean curve of BMP2 expression of 291 patients with different HB grades (Fig. 7).

**Figure 7:**
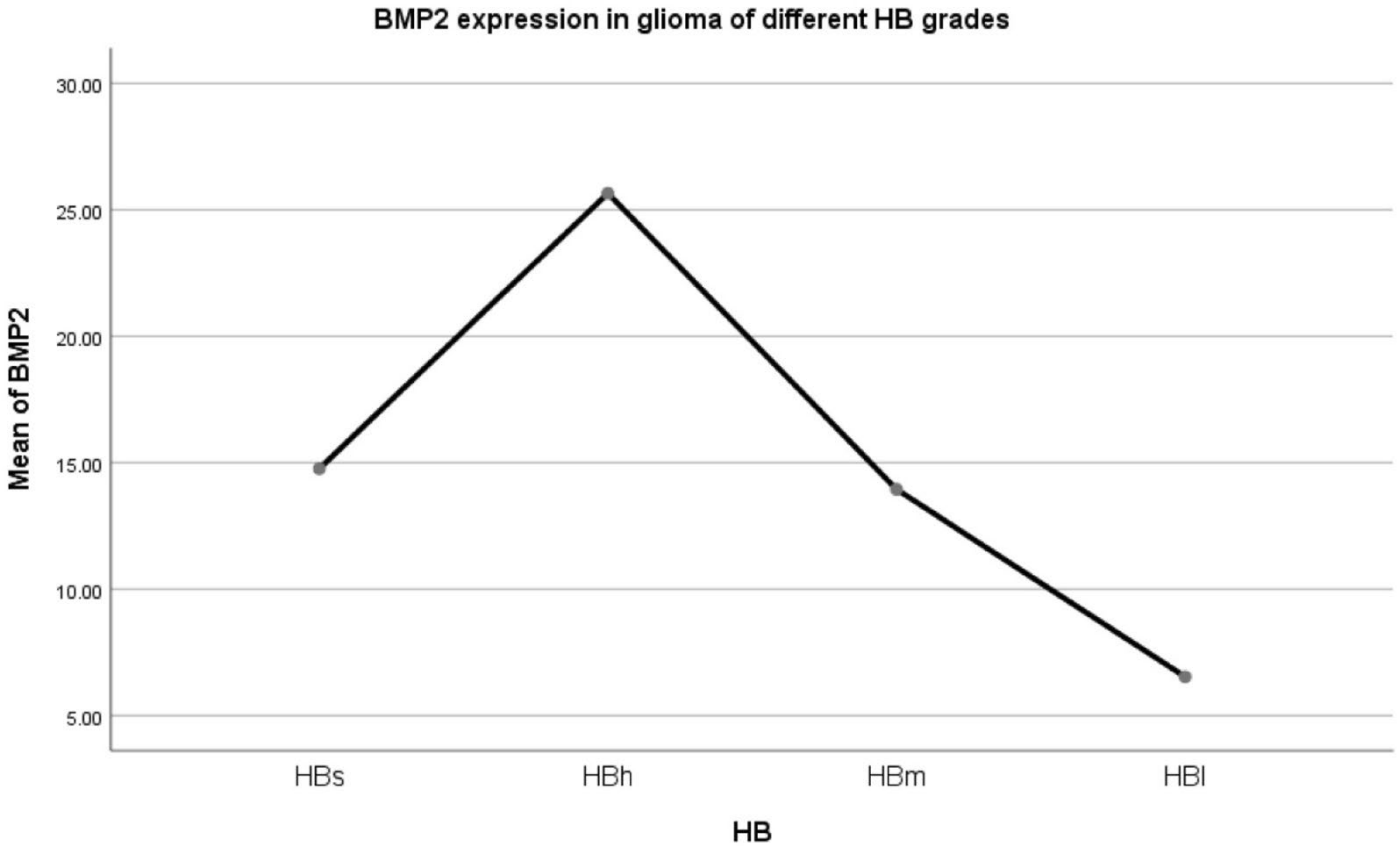
Mean curve of Bone Morphogenetic Protein 2 (BMP2) mRNA expression of 291 patients with different HB grades using the new HB grading system.
    There were only three cases of HBs, so there was no statistical difference to be further verified. The other three groups had statistical significance and the trend was consistent with the model (F = 29.089, *p* = 0.000). Pairwise Comparison noted that there were significant differences (*p* = 0.000). HBh > HBm > HBl.
  1.4 Survival analysis with different HB grade glioma in the CGGA validation group Survival analysis function (Fig. 8).

**Figure 8:**
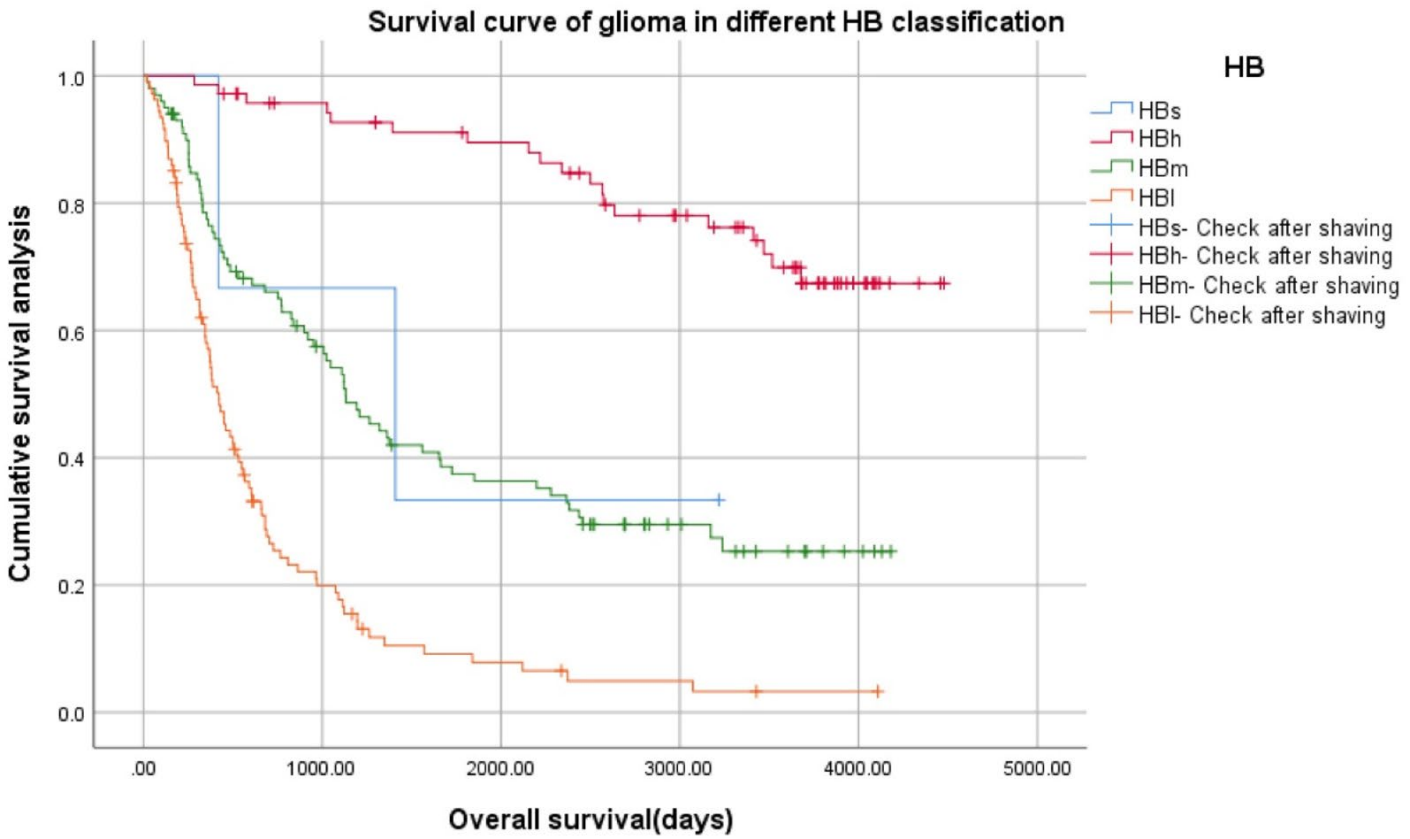
Survival analysis using the different HB grade glioma in the Chinese Glioma Genome Atlas (CGGA) validation group Survival analysis function.
    Log Rank (Mantel–Cox) x^2^ = 130.379, *p* = 0.000 and the trend was consistent with the model. 10 cases with incomplete follow-up data were excluded from 291 cases, therefore, 3 cases of HBs, 71 cases of HBh, 100 cases of HBm, 107 cases of HBl, totaling 281 cases.
2. Validation group 2: 625 patients from TCGA database
  2.1 General condition of 625 glioma patients with different histopathological types in the TCGA verification group (Table 5; Fig. 9).

**Table 5:** Clinical characteristics of 625 patients.

Variable	n	Age (year)	IDH1 mutation (yes/no/NA)	1p19q LOH (yes/no/NA)	Mean BMP2
O	174	45.50 ± 1.10	NA	NA	10.34 ± 0.11
OA	127	41.11 ± 1.19	NA	NA	9.96 ± 0.12
A	180	42.02 ± 0.94	NA	NA	9.24 ± 0.12
GBM	144	59.97 ± 1.10	NA	NA	7.79 ± 0.09
Total	625	46.94 ± 0.60	NA	NA	9.36 ± 0.07

**Figure 9:**
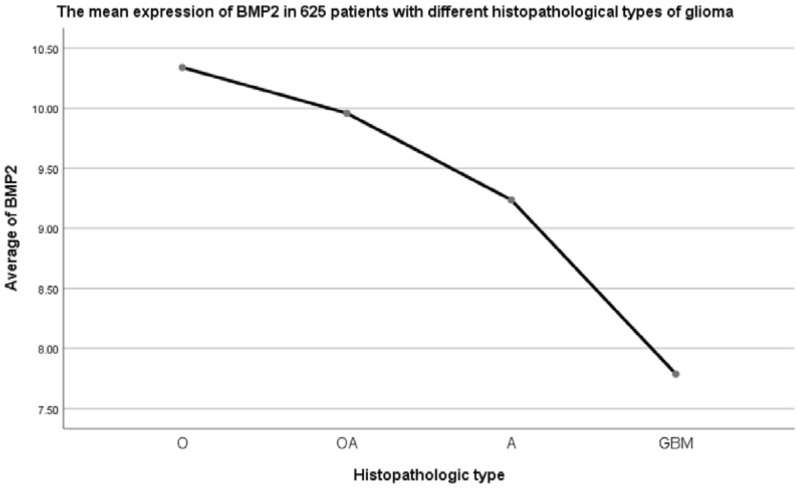
Bone Morphogenetic Protein 2 (BMP2) mRNA expression was different in patients with different histopathological types (F = 94.391, *p* = 0.000).
    Patients from TCGA database were divided into four types according to histopathological characteristics: O, OA, A and GBM, but IDH1 and 1p19q LOH information were not provided. There was a difference in BMP2 mRNA expression among four groups, in which GBM was higher than other three groups (F = 64.632, *p* = 0.000). In pairwise comparison, the differences between the two types were statistically significant (pO-OA = 0.022, pO-A = 0.000, pO-GBM = 0.000, pOA-A = 0.000, pOA-GBM = 0.000, pA-GBM = 0.000). O > OA > A > GBM.
  2.2 Clinical characteristics of 625 patients based on HB classification (Table 6; Fig. 10).

**Table 6:** Clinical characteristics of 625 patients based on HB classification.

HB	n	Age (year)	IDH1 mutation (yes/no/NA)	1p19q LOH (yes/no/NA)	Mean BMP2
HBs	0	0	0	0	0
HBh	301	43.64 ± 0.78	NA	NA	10.18 ± 0.08
HBm	180	42.02 ± 0.94	NA	NA	9.24 ± 0.12
HBl	144	59.97 ± 1.10	NA	NA	7.79 ± 0.09
Total	625	46.94 ± 0.60	NA	NA	9.36 ± 0.07

**Figure 10:**
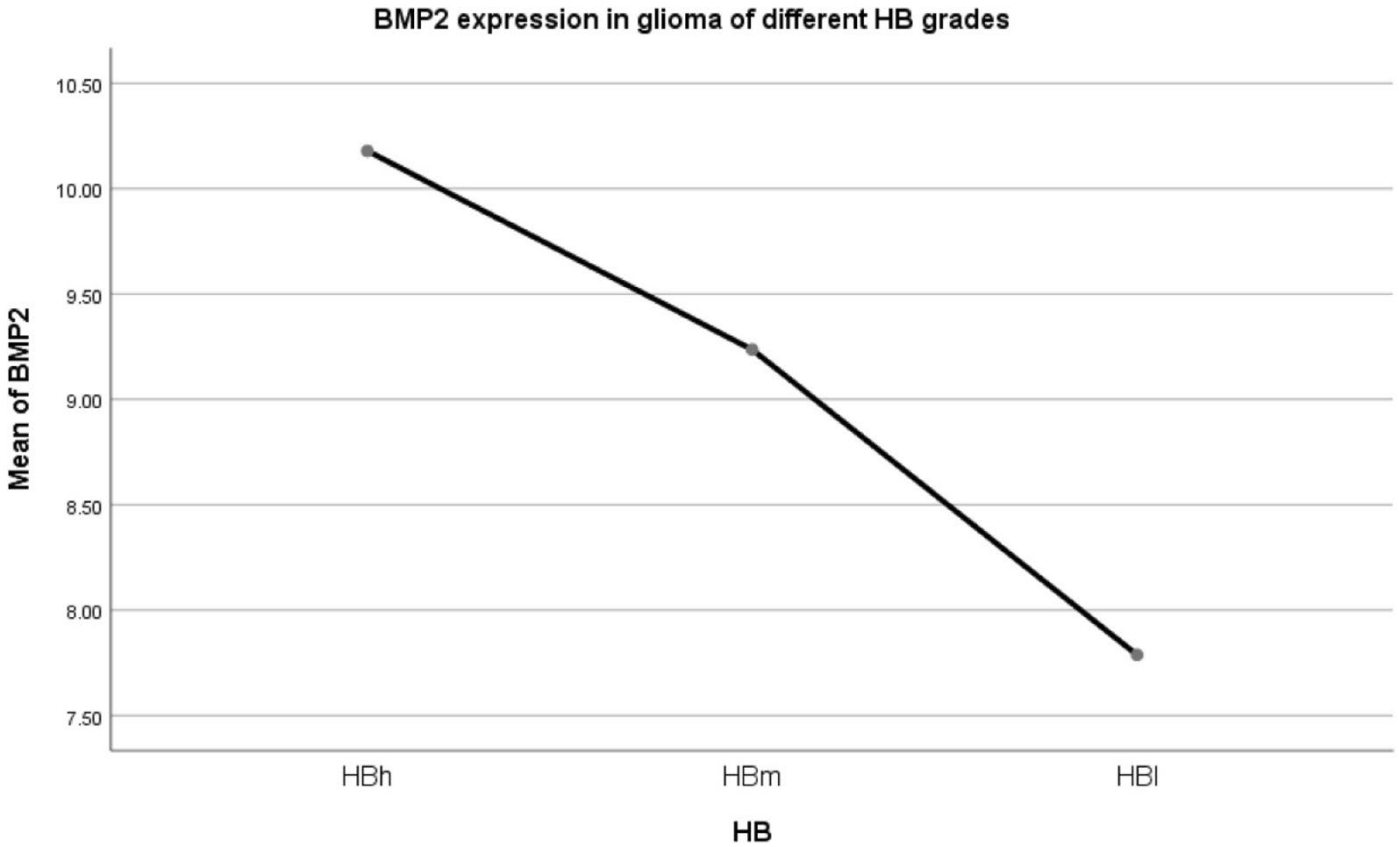
Bone Morphogenetic Protein 2 (BMP2) mRNA expression was different in patients with different histopathological types defined by the proposed HB grading system (F = 138.000, *p* = 0.000).
    In pairwise comparison, there were statistically significant differences between the two types (*p* = 0.000). HBh > HBm > HBl.
  2.3 Survival analysis of 625 patients with different HB grade gliomas in TCGA validation group (Fig. 11).

**Figure 11:**
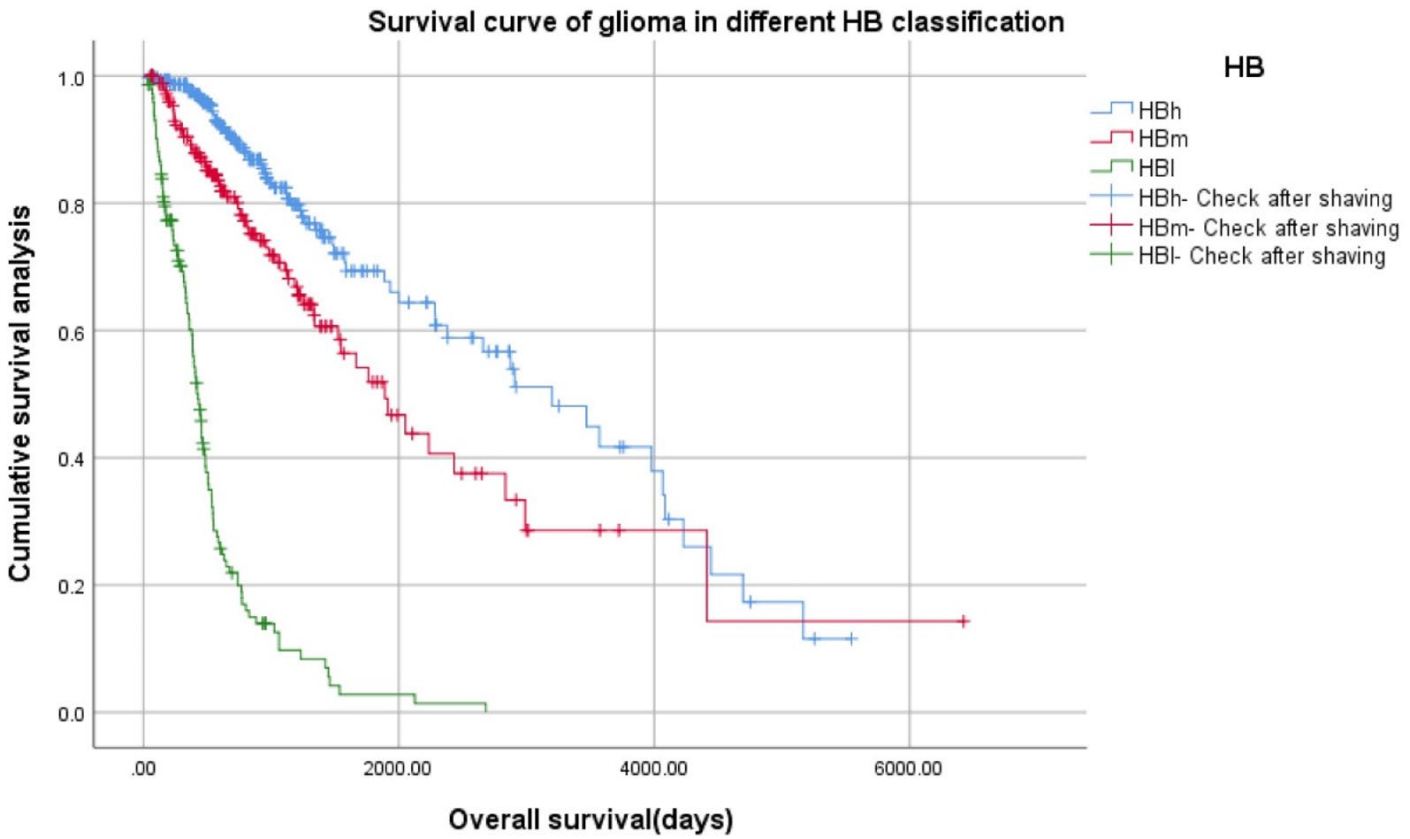
Survival analysis of 625 patients with different HB grade gliomas in The Cancer Genome Atlas (TCGA) validation group.
    Log Rank (Mantel–Cox) x^2^ = 340.716, *p* = 0.000, the survival curve trend was completely consistent with the model.

### Model extension application

1. Expression of BMP2 receptor 1A (BMPR1A) in different HB grade gliomas (Fig. 12).

**Figure 12:**
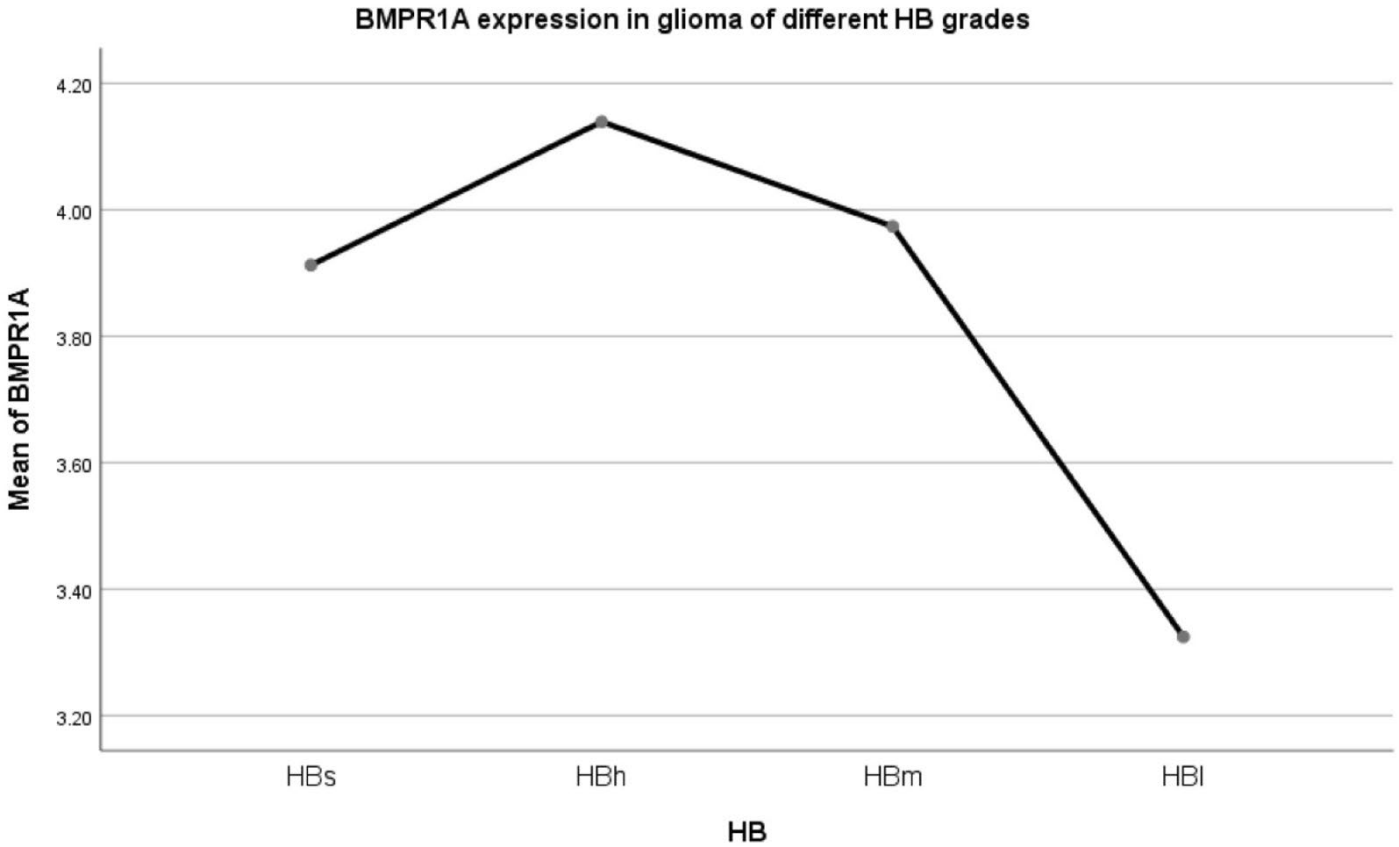
Mean BMPR1A expression within patients with different HB grade glioma.
  The expression levels of BMPR1A in HBs/HBh/HBm were not statistically significant, and the expression levels in HBl group were lower than HBh and HBm. F = 3.799, *p* = 0.010. LSD method pairwise comparison: pHBs-HBh = 0.614, pHBs-HBm = 0.883, pHBs-HBl = 0.161, pHBh-HBm = 0.559, pHBh-HBl = 0.005, pHBm-HBl = 0.005.
2. Expression of BMP2 receptor 2 (BMPR2) in different HB grade gliomas (Fig. 13).

**Figure 13:**
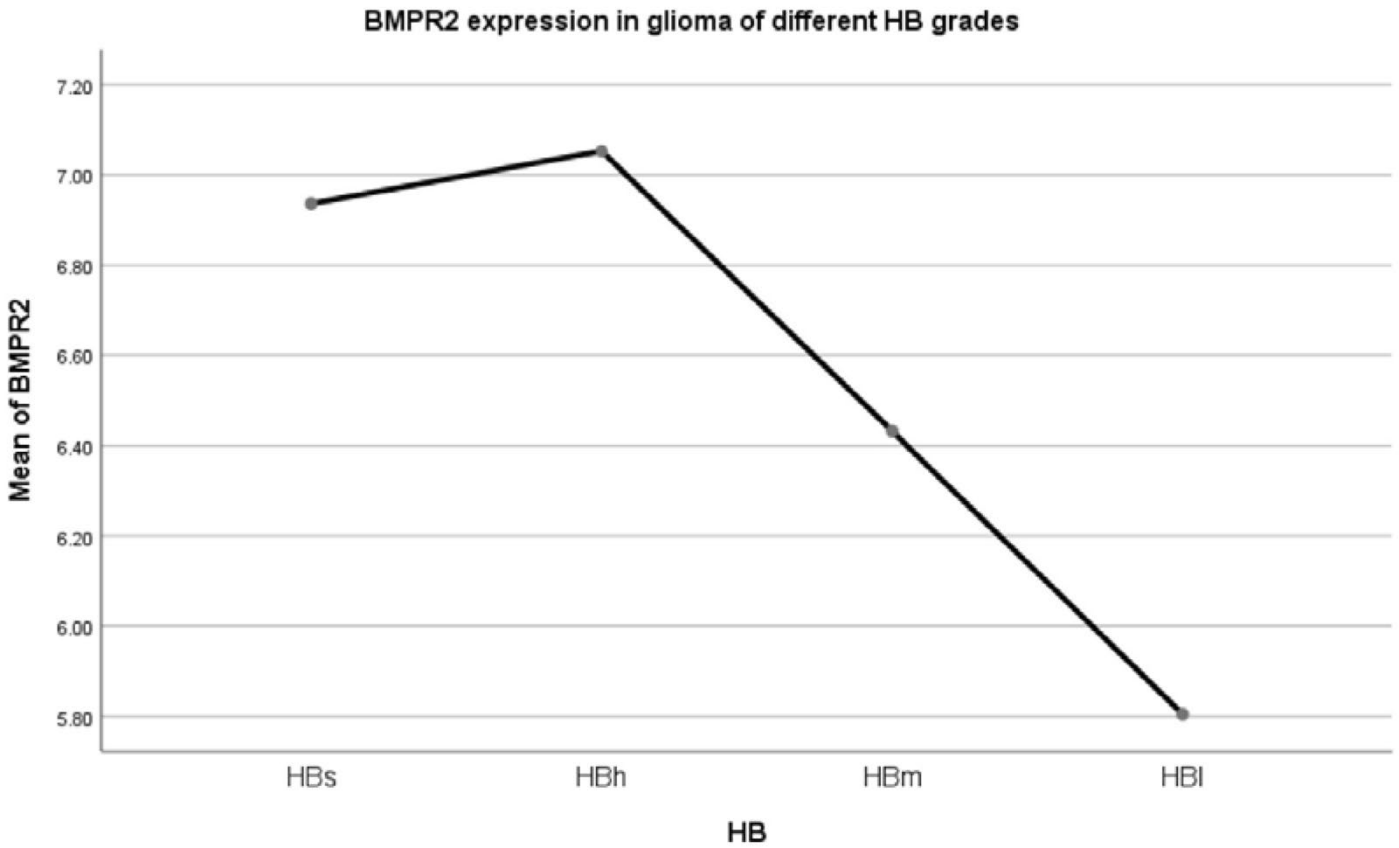
Mean BMPR2 expression of patients with different HB grade glioma.
  The expression level of BMPR2 in HBs/HBh/HBm was not statistically significant. The expression level of the HB1 group was lower than that of the above three groups, F = 4.540, *p* = 0.004. LSD method pairwise comparison: pHBs-HBh = 0.840, pHBs-HBm = 0.345, pHBs-HBl = 0.035, pHBh-HBm = 0.087, pHBh-HBl = 0.001, pHBm-HBl = 0.035.
3. Expression of BMP2 receptor 1B (BMPR1B) in different HB grade gliomas (Fig. 14).

**Figure 14:**
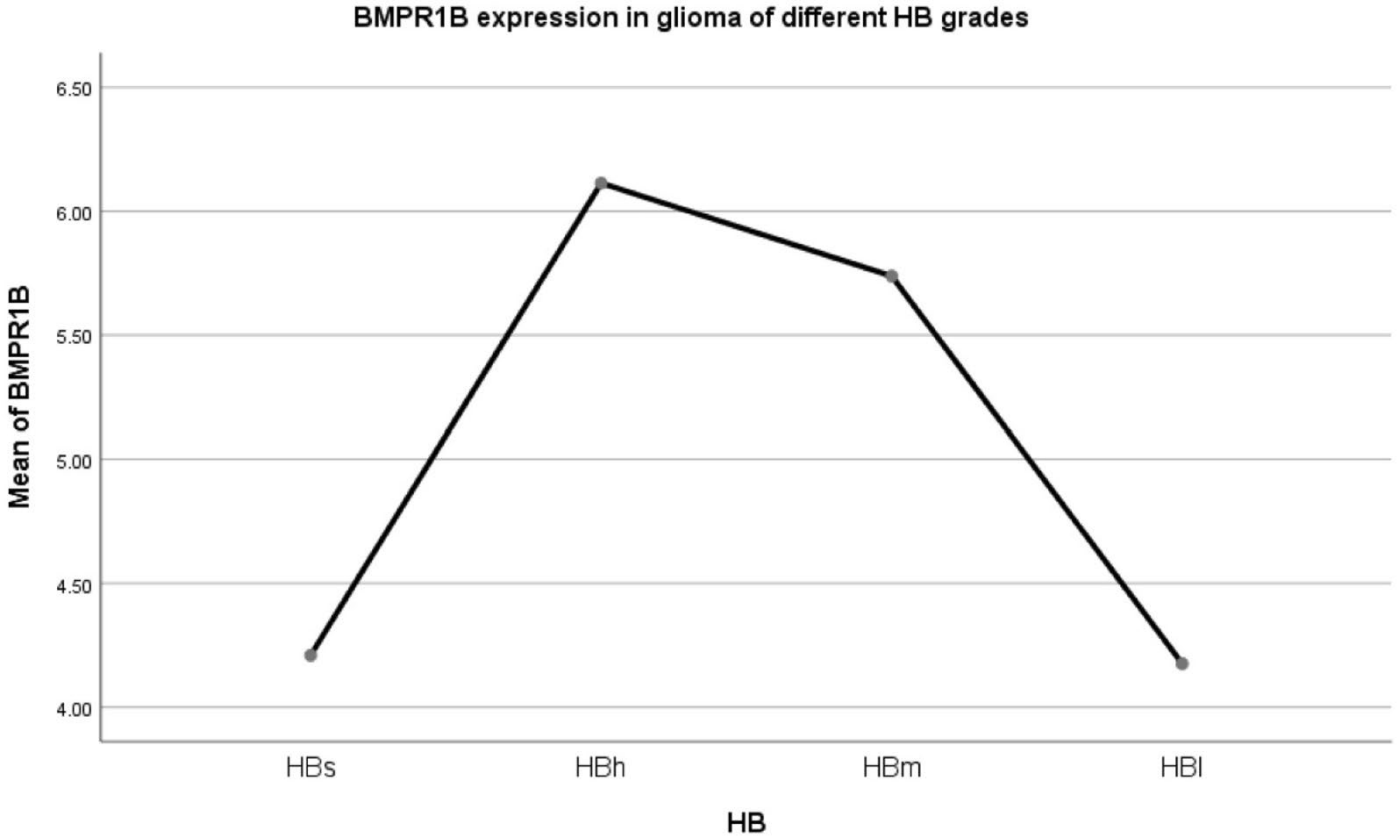
Mean BMPR1B expression within patients with different HB grade glioma.
  The expression level of BMPR1B in HBs decreased significantly, and the difference was statistically significant, similar to the HB1 group. There was no difference in HBh/HBm expression levels. F = 9.736, *p* = 0.010. LSD method pairwise comparison: pHBs-HBh = 0.007, pHBs-HBm = 0.018, pHBs-HBl = 0.958, pHBh-HBm = 0.394, pHBh-HBl = 0.000, pHBm-HBl = 0.000.
4. Summary of the expression of the above three BMPRs (Table 7).

**Table 7:** BMP2/BMPR1A/BMPR2/BMPR1B expression and its relationship with survival in 692 glioma patients with different HB types in CGGA.

HB classification	BMP2	BMPR1A	BMPR2	BMPR1B	Survival
HBs	Extremely high	High	High	Low	Middle
HBh	High	High	High	High	Good
HBm	Middle	High	High	High	Middle
HBl	Low	High	High	Low	Difference

  4.1 In the HBs group, the expression of BMP2 was very high, the expression of BMPR1A/BMPR2 was high, but the expression of MPR1B was low, which led to the middle prognosis.
  4.2 In the HBh group, the expression of BMP2 and BMPR1A/BMPR2/BMPR1B were high, and the prognosis was good.
  4.3 In the HBm group, the expression of BMP2 was moderate, although the expression of BMPR1A/BMPR1B/BMPR2 was high, the prognosis was affected by the moderate expression of BMP2, and the prognosis was moderate.
  4.4 In the HBl group, the expression of BMP2 was low, although the expression of BMPR1A/BMPR2 was high, the expression of BMPR1B was low, and the prognosis was poor.

## Discussion

### BMP system

BMPs belong to the TGF-β super family, which are the regulatory factors for the dynamic and balanced growth of pluripotent stem cells^14^. They play an important role in the growth and development of the body by coordinating the differentiation, proliferation and apoptosis of cells in different tissues and organs^15–18^. Recent studies have shown that BMPs play an important role in tumor cell proliferation, metastasis, angiogenesis and differentiation^19–21^. Within the BMP family, human BMP2 cDNA has a total length of 1587 bp, encoding about 396 amino acids, and its N-terminal untranslated region is about 340 bp in length. It is a glycoprotein composed of homologous dimers with a molecular weight of 32 kD and each monomer contains 114 amino acids.

### BMPR system

BMP receptors (BMPRs) are widely expressed in many tissues and cells of the human body. BMP activates downstream signals by binding to type I and type II receptors on the cell membrane, both of which co-transduce BMP signals. Among them, BMPR1A, BMPR1B and BMPR2 are specific subtypes of BMPRs.

### Mechanism of action of BMP2

During signal transduction, the BMP2 dimer forms a stable complex structure with type I and type II receptors. Type I receptors are responsible for signal transduction, whereas type II receptors phosphorylate type I. This phosphorylation allosterically activates the intracellular serine/threonine kinase domain of type I receptor. When BMPs bind to the complex, the type II receptor phosphorylates the glycine-serine (GS) region of the type I receptor, causing a conformational change and thus activation of the type I receptor, which in-turn activates the Receptor-associated SMAD (R-SMAD) protein. Following activation, the R-SMAD protein can form heterologous complexes with Smad4 in the cytoplasm and enter the nucleolus through the MH2 domain to regulate the transcription of target genes^22–25^. During development, the expression of BMPR transited from fetal type (BMPR1A) which induced the proliferation of neuroepithelial cells to adult type receptor type (BMPR1B) which mediated the differentiation of astrocytes and neurons. The differentiation characteristics of BMPs make them potential candidates for GBM therapy^26–29^. In addition, due to the higher affinity of type I receptor to BMPs than type II receptor, when BMPs first bind to type I receptor, the independent smad signal transduction pathway is activated. MAPK pathway was activated to induce its action^30–32^.

At present, it has been found that BMPs play a double-edged role in tumor biology, and its effect on tumor inhibition or promotion depends on the tissue type and microenvironment of BMPs. Steinert et al.^33^ found through in vitro experiments that: in breast cancer cells with normal nutrition, BMP2 can promote the occurrence of apoptosis by transcription of apoptosis-related genes (such as PKR, EIF2α); in breast cancer cells with malnutrition, BMP2 can generate ID-1 through the MAPK signal transduction pathway and inhibit the activation of Caspase-3 to enhance the viability of breast cancer cells. This phenomenon suggests that there should be some type of cell homeostasis mechanism in the process of tumor morphogenesis to regulate the induction or inhibition mediated by BMP^11,34^.

We established a new glioma classification model based on histochemistry and BMP2 expression (HB classification: HBs: rO, rAO, rOA; HBh: O, AO, OA; HBm: AOA, rAOA, A, rA, AA, rAA; HBl: GBM, rGBM), which is related to the prognosis of patients. This grading system is slightly different from that used by the WHO, for example in that rO, rAO and rOA in HBs group have a worse prognosis. WHO classified rO and rOA as level II, while in our grading system, we found that their adverse survival prognosis is similar to that of AOA, rAOA, A, rA, AA and rAA. There was no difference in the survival time of O, AO and OA in the HBh group, suggesting that the biological behavior of these patients might be similar, suggesting that AO has a better prognosis, while WHO classified it as level III. rA has poor prognosis in HBm group, but WHO classified it as grade II.

In gliomas, BMPR1A and BMPR2 were highly expressed in different types of gliomas, but there was no significant difference between them. The expression of BMP2 and BMPR1B are different in different types of gliomas, and their different combinations lead to different prognosis of different types of gliomas. It is suggested that BMP2 and BMPRIB are the key factors to promote the apoptosis and differentiation of tumors and lead to different types of gliomas with different prognosis.

The effect of BMP2 on glioma differentiation and apoptosis is receptor dependent, which mainly depends on the receptor BMPR1B. The higher the expression of BMPR1B, the better the prognosis. Liu Shuang et al. showed that the overexpression of BMPR1B significantly inhibited the growth of glioma cells and promoted the differentiation of glioma cells. In the animal model system, the over expression of BMPR1B significantly inhibited the tumorigenicity of glioblastoma cells, while the decreased expression of BMPR1B significantly enhanced the tumorigenicity of glioblastoma cells. The overexpression of BMPR1B activated the BMPs/Smad1/5/8 signaling pathway and inhibited the growth of glioma cells through various mechanisms, including the decrease of SKP2 expression, followed by the increase of p21 and p27kip1 Protein Expression^35^.

BMP2 increased the differentiation and apoptosis of glioma in a concentration dependent manner. In the case of high expression of BMPR1B, the higher the concentration of BMP2, the better the prognosis. In the company of low BMPR1B expression, BMP2 increased the differentiation and apoptosis of glioma in a concentration dependent manner. The lower the concentration, the worse the prognosis.

### The clinical significance of this study

For HBs patients, in order to improve the survival period of patients, we should consider improving the expression level of BMPR1B, rather than the expression level of BMP2, because the main manifestation is high expression of BMP2 and low expression of BMPR1B. For HBm patients, we should focus on increasing the expression of BMP2, because it is mainly the high expression of BMPR1B and medium expression of BMP2 that occurs. For HBl patients, the expression of BMP2 and BMPR1B should be increased at the same time, because the expression of them in HBl patients is very low.

## Methods

### Model establishment

The clinical information, histopathological results, survival time, and BMP2 mRNA expression level of 692 patients were extracted from the Chinese Glioma Genome Atlas (CGGA) database. Their clinical characteristics, histopathological and molecular pathological results, and the WHO classification were approved by the institutional review committee of Beijing Tiantan Hospital (Beijing, China). Informed consent was obtained from all subjects or, if subjects are under 18, from a parent and/or legal guardian. All experimental methods were carried out in accordance with the relevant guidelines and regulations. The WHO categorizes human gliomas by grade, each of which is subdivided into different types, for example grade II consists of type A, O, OA, rA and rOA. According to this system and the presentation of defining histopathological characteristics, patients were divided into 14 groups: O, rO, AO, rOA, OA, rOA, AOA, rAOA, A, rA, AA, rAA, GBM, and rGBM. The expression of BMP2 mRNA within these 14 types of glioma was statistically analyzed. We named the new classification based on both histopathological characteristics and BMP2 mRNA expression as histopathologic-BMP2 (HB) classification. The patients were also further divided into four grades—HBs, HBh, HBm, and HBl in accordance with the varying level of expression of BMP2 mRNA. Among them, HBs includes rO, rAO, and rOA; HBh includes O, AO, and OA; HBm includes AOA, rAOA, A, rA, AA, and rAA; HBl includes GBM and rGBM. Additionally, we analyzed the survival period of patients with gliomas.

### Model validation

The clinical information, histopathological results, survival time and BMP2 mRNA expression of 291 patients in another group and of 625 patients were collected from CGGA database and TCGA database, respectively. We performed BMP2 expression and survival analysis to evaluate the validity of the results.

### Extended application

The expression of BMPR1A/BMPR1B/BMPR2 in four grades of gliomas was detected based on HB classification model. Meanwhile, the expression of BMP2 was comprehensively analyzed to gain further insight into the mechanism of action of BMP2.

### Statistical analysis

We used one-way analysis of variance (ANOVA) in SPSS v25.0 statistical software for the expression of BMP2 mRNA and BMPR1A, BMPR1B as well as the BMP2 receptor (BMPR2) mRNA in patients with different grades of gliomas. Least-significant difference (LSD) method was then used for comparison. Independent sample t test was used to compare the difference of BMP2 mRNA expression between the two groups. Moreover, Kaplan–Meier curve was calculated to determine the survival period of patients, and log-rank analysis was used to determine the significance. In this study, the data were shown as mean ± SD. All statistical tests were bidirectional and *p* < 0.05 was considered statistically significant.

## Conclusion

The role of BMP2 in promoting tumor differentiation and apoptosis in different types of gliomas is mainly attributed to its BMPR1B receptor dependence and its own concentration dependence. If both are highly expressed, the prognosis of the patient is good. If either is decreased, the prognosis of patient will be worse. The expression of both depends on the difference of pathological components and the change of microenvironment. How from the gene level, around the tumor microenvironment change, and the upstream and downstream of BMP2/BMPR shaft signal regulation factor analytic BMP2 and BMPR1B receptor expression in different tissue pathology of different causes and mechanism, the molecular mechanism of organization to maintain dynamic balance of BMP2, BMP2/BMPR shaft and other non BMPR/BMP2/shaft of signaling pathways, artificial synthesis of BMP2 clinical application needs further study. Our HB classification model establishes a meaningful classification model for the follow-up study.
